# *rac*-Eth­yl(phen­yl)phosphinic acid

**DOI:** 10.1107/S160053681204812X

**Published:** 2012-11-30

**Authors:** Robert A. Burrow, Rubia M. Siqueira da Silva

**Affiliations:** a Laboratório de Materiais Inorgânicos, Universidade Federal de Santa Maria, 97105-900 Santa Maria–RS, Brazil

## Abstract

The crystal structure of the title compound, C_8_H_11_O_2_P, features O—H⋯O hydrogen bonds, which link mol­ecules related by the *b*-glide plane into chains along [010].

## Related literature

For background to metal-organic frameworks involving phospho­nate ligands, see: Gagnon *et al.* (2012 ▶). For details of coordination polymers constructed using phosphinic acids as the spacer ligand, see: Siqueira *et al.* (2006 ▶); Beckmann *et al.* (2009 ▶). For further details of phosphinic acids and the crystal structures of similar compounds, see: Burrow *et al.* (2000 ▶); Burrow & Siqueira da Silva (2011*a* ▶,*b* ▶). For a description of the Cambridge Structural Database, see: Allen (2002 ▶). For geometry analysis using *Mogul*, see: Bruno *et al.* (2004 ▶).
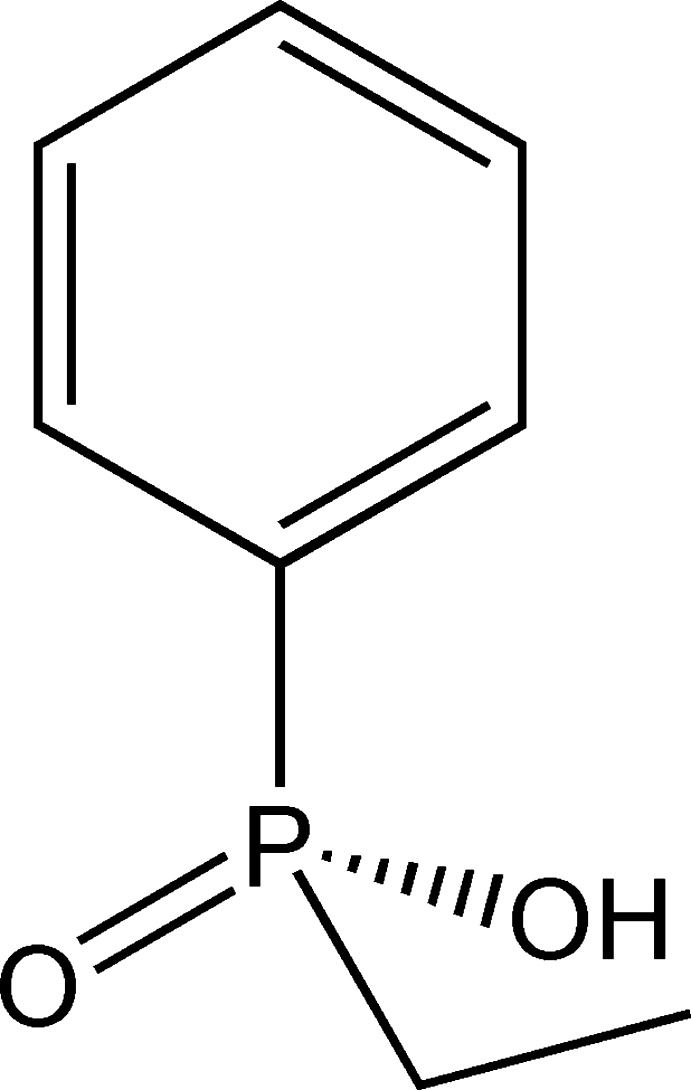


## Experimental

### 

#### Crystal data

C_8_H_11_O_2_P
*M* *_r_* = 170.14
Orthorhombic, 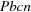

*a* = 13.5314 (16) Å
*b* = 8.0328 (9) Å
*c* = 15.922 (2) Å
*V* = 1730.6 (4) Å^3^
*Z* = 8
Mo *K*α radiation
μ = 0.27 mm^−1^
*T* = 296 K
0.41 × 0.12 × 0.11 mm

#### Data collection

Bruker X8 Kappa APEXII diffractometer
Absorption correction: multi-scan (*SADABS*; Bruker, 2012 ▶) *T*_min_ = 0.906, *T*_max_ = 0.971
14069 measured reflections
2650 independent reflections
1499 reflections with *I* > 2σ(*I*)
*R*_int_ = 0.054

#### Refinement

*R*[*F* ^2^ > 2σ(*F* ^2^)] = 0.045
*wR*(*F* ^2^) = 0.128
*S* = 1.09
2650 reflections
104 parameters
H atoms treated by a mixture of independent and constrained refinement
Δρ_max_ = 0.24 e Å^−3^
Δρ_min_ = −0.34 e Å^−3^

### 

Data collection: *APEX2* (Bruker, 2012 ▶); cell refinement: *SAINT* (Bruker, 2012 ▶); data reduction: *SAINT*; program(s) used to solve structure: *SHELXS97* (Sheldrick, 2008 ▶); program(s) used to refine structure: *SHELXL97* (Sheldrick, 2008 ▶); molecular graphics: *DIAMOND* (Brandenburg, 2012 ▶); software used to prepare material for publication: *SHELXL97* and *publCIF* (Westrip, 2010 ▶).

## Supplementary Material

Crystal structure: contains datablock(s) global, I. DOI: 10.1107/S160053681204812X/su2532sup1.cif

Structure factors: contains datablock(s) I. DOI: 10.1107/S160053681204812X/su2532Isup2.hkl

Supplementary material file. DOI: 10.1107/S160053681204812X/su2532Isup3.cdx

Supplementary material file. DOI: 10.1107/S160053681204812X/su2532Isup4.cml

Additional supplementary materials:  crystallographic information; 3D view; checkCIF report

## Figures and Tables

**Table 1:** Hydrogen-bond geometry (Å, °)

*D*—H⋯*A*	*D*—H	H⋯*A*	*D*⋯*A*	*D*—H⋯*A*
O1—H1⋯O2^i^	0.87 (2)	1.64 (2)	2.4931 (19)	168 (2)

Symmetry code: (i) 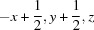.

**Fig. 1.**
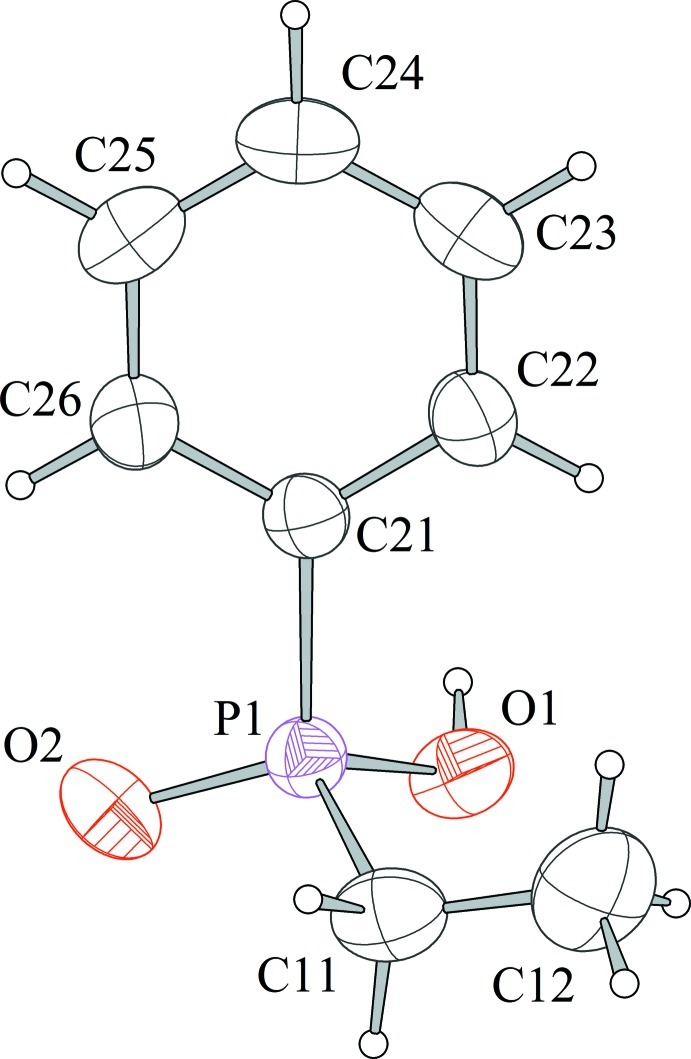
The molecular structure of the title molecule, with the atom numbering. The displacement ellipsoids are drawn at the 50% probability level.

**Fig. 2.**
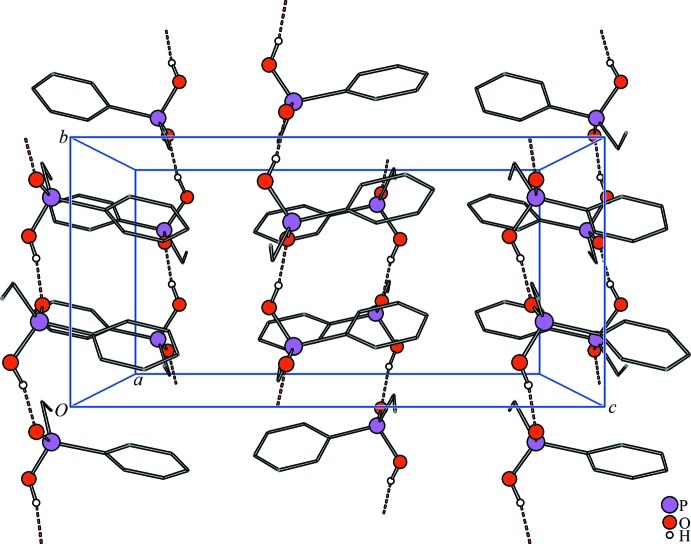
A perspective view along the a axis of the crystal packing of the title compound. The O-H···O hydrogen bonds are shown as dashed lines.

## supplementary crystallographic information

### Comment

Coordination polymers are the basis of metal-organic frameworks usually based on carboxylate ligands or phosphonate ligands (Gagnon *et al.*, 2012). Coordination polymers have also been constructed using phosphinic acids as the spacer ligand (Siqueira *et al.*, 2006; Beckmann *et al.*, 2009). Continuing our research on phosphinic acids (Burrow *et al.*, 2000; Burrow & Siqueira da Silva, 2011*a*,*b*), we report herein on the synthesis and crystal structure of the title compound.

The title compound, Fig. 1, is found to crystallize as a racemic mixture of enantiomers in the centrosymmetric space group Pbcn. An analysis of the geometry with Mogul [Bruno *et al.*, 2004] using the Cambridge Structural Database [CSD; Allen, 2002] showed a slightly wider C—P—C angle [110.87 (9) °] than average [mean = 106.0(2.2)° of 15 observations] with |z-score| = 2.178. The P—O distance, though not unusual at 1.5529 (14) Å, is slightly longer than average value [mean 1.542 (22) Å of 17 observations] and is similar to that in methyl(phenyl)phosphinic acid [1.5526 (16) Å; Burrow & Siqueira da Silva, 2011*b*].

In the crystal, hydrogen bonding interactions (Table 1 and Fig. 2) of the type OH···O=P—OH···O=P join molecules related by the *b* glide plane into continuous chains along [010]. The short P—O···O=P distance of 2.4931 (19) Å indicates a strong hydrogen bond. This is slightly shorter than the average O···O interaction distance in the CSD [2.51 (5) Å of 60 observations] for other phosphinic acids, but is equal that for methyl(phenyl)phosphinic acid, 2.4838 (18) Å [Burrow & Siqueira da Silva, 2011*b*].

The crystal packing diagram, Fig. 2, shows that the hydrogen bonded chains of the title compound form columns in the crystallographic b direction, with the chains alternating direction in the other two dimensions. There are no phenyl-phenyl interactions.

### Experimental

To a solution of phenylphosphinic acid (2.0 g, 14.1 mmol) in dichloromethane, diisopropylethylamine (5.16 ml, 29.6 mmol) and trimethylsilyl chloride (3.74 ml, 29.6 mmol) were separately added at 273 K under argon. The reaction mixture was stirred at room temperature for 2–3 h, cooled to 273 K and ethyliodide (1.25 ml, 19.6 mmol) was added. After further stirring at room temperature for 48 h, the solvent was removed under vacuum. The residue was suspended in hydrochloric acid (2 *M*, 20 ml) and filtered on a glass frit. The white solid was washed with acetone and dried giving a yield of 0.84 g (35%) of pure product. Crystals suitable for single-crystal X-ray analysis were grown from an acetone solution in a desiccator with silica gel. Spectroscopic and TGA data for the title compound are available in the archived CIF.

### Refinement

The H atom on O1 was located in a difference Fourier map and its position was allowed to refine freely with U_iso_(H) = 1.5 U_eq_(O). The C-bound H atoms were positioned geometrically and allowed to ride on their parent atoms: C—H = 0.93, 0.97 and 0.97 Å for CH, CH_2_ and CH_3_ H atoms, respectively, with = k × U_eq_(C), where k = 1.5 for CH_3_ H atoms and = 1.2 for other H atoms.

### Crystal data

**Table:**

C_8_H_11_O_2_P	*D*_x_ = 1.306 Mg m^−^^3^
*M**_r_* = 170.14	Melting point = 336–341 K
Orthorhombic, *P**b**c**n*	Mo *K*α radiation, λ = 0.71073 Å
*a* = 13.5314 (16) Å	Cell parameters from 2263 reflections
*b* = 8.0328 (9) Å	θ = 2.6–26.5°
*c* = 15.922 (2) Å	µ = 0.27 mm^−^^1^
*V* = 1730.6 (4) Å^3^	*T* = 296 K
*Z* = 8	Block, colourless
*F*(000) = 720	0.41 × 0.12 × 0.11 mm

### Data collection

**Table:**

Bruker X8 Kappa APEXII diffractometer	2650 independent reflections
Radiation source: sealed ceramic X ray tube, Siemens KFF	1499 reflections with *I* > 2σ(*I*)
Graphite crystal monochromator	*R*_int_ = 0.054
Detector resolution: 8.3333 pixels mm^-1^	θ_max_ = 30.5°, θ_min_ = 3.2°
0.5 ° ω & φ scans	*h* = −19→19
Absorption correction: multi-scan (*SADABS*; Bruker, 2012)	*k* = −9→11
*T*_min_ = 0.906, *T*_max_ = 0.971	*l* = −18→22
14069 measured reflections	

### Refinement

**Table:**

Refinement on *F*^2^	Primary atom site location: structure-invariant direct methods
Least-squares matrix: full	Secondary atom site location: difference Fourier map
*R*[*F*^2^ > 2σ(*F*^2^)] = 0.045	Hydrogen site location: inferred from neighbouring sites
*wR*(*F*^2^) = 0.128	H atoms treated by a mixture of independent and constrained refinement
*S* = 1.09	*w* = 1/[σ^2^(*F*_o_^2^) + (0.0525*P*)^2^ + 0.0622*P*] where *P* = (*F*_o_^2^ + 2*F*_c_^2^)/3
2650 reflections	(Δ/σ)_max_ < 0.001
104 parameters	Δρ_max_ = 0.24 e Å^−^^3^
0 restraints	Δρ_min_ = −0.34 e Å^−^^3^

### Special details

**Table:**

Experimental. Spectroscopic and TGA data for the title compound:IR: 1438 (*m*), 1177 (*versus*), 1137 (*s*), 998 (*versus*), 935 (*versus*), 745 (*m*), 718 (*s*), 692 (*versus*), 565 (*m*), 537 (*m*), 495 (*m*) cm^-1^. TGA: 483 - 603 K; 88% loss.
Geometry. All e.s.d.'s (except the e.s.d. in the dihedral angle between two l.s. planes) are estimated using the full covariance matrix. The cell e.s.d.'s are taken into account individually in the estimation of e.s.d.'s in distances, angles and torsion angles; correlations between e.s.d.'s in cell parameters are only used when they are defined by crystal symmetry. An approximate (isotropic) treatment of cell e.s.d.'s is used for estimating e.s.d.'s involving l.s. planes.
Refinement. Refinement of *F*^2^ against ALL reflections. The weighted *R*-factor w*R* and goodness of fit *S* are based on *F*^2^, conventional *R*-factors *R* are based on *F*, with *F* set to zero for negative *F*^2^. The threshold expression of *F*^2^ > 2σ(*F*^2^) is used only for calculating *R*-factors(gt) *etc*. and is not relevant to the choice of reflections for refinement. *R*-factors based on *F*^2^ are statistically about twice as large as those based on *F*, and *R*-factors based on ALL data will be even larger.

### Fractional atomic coordinates and isotropic or equivalent isotropic displacement parameters (Å^2^)

**Table:**

	*x*	*y*	*z*	*U*_iso_*/*U*_eq_	
P1	0.31872 (4)	0.19541 (6)	0.40958 (3)	0.03859 (18)	
O1	0.34640 (11)	0.35269 (17)	0.35798 (8)	0.0468 (4)	
H1	0.3212 (16)	0.444 (3)	0.3780 (15)	0.07*	
O2	0.21554 (10)	0.13429 (17)	0.39751 (8)	0.0496 (4)	
C11	0.40682 (16)	0.0441 (3)	0.37446 (13)	0.0539 (5)	
H11A	0.4004	−0.0541	0.4095	0.065*	
H11B	0.3897	0.012	0.3176	0.065*	
C12	0.51433 (18)	0.0978 (3)	0.37521 (16)	0.0740 (7)	
H12C	0.5222	0.1953	0.341	0.111*	
H12A	0.5547	0.0096	0.3533	0.111*	
H12B	0.5341	0.1224	0.4318	0.111*	
C21	0.33743 (13)	0.2444 (2)	0.51841 (11)	0.0368 (4)	
C22	0.41968 (14)	0.3314 (2)	0.54585 (13)	0.0474 (5)	
H22	0.4675	0.3645	0.5073	0.057*	
C23	0.43146 (16)	0.3697 (3)	0.62986 (14)	0.0584 (6)	
H23	0.4868	0.4286	0.6476	0.07*	
C24	0.36130 (17)	0.3207 (3)	0.68740 (14)	0.0579 (6)	
H24	0.3694	0.3464	0.7439	0.069*	
C25	0.28001 (17)	0.2344 (3)	0.66167 (13)	0.0576 (6)	
H25	0.2329	0.2012	0.7008	0.069*	
C26	0.26725 (15)	0.1960 (2)	0.57756 (12)	0.0466 (5)	
H26	0.2115	0.1374	0.5605	0.056*	

### Atomic displacement parameters (Å^2^)

**Table:**

	*U*^11^	*U*^22^	*U*^33^	*U*^12^	*U*^13^	*U*^23^
P1	0.0484 (3)	0.0289 (3)	0.0386 (3)	0.0015 (2)	−0.0015 (2)	0.0000 (2)
O1	0.0652 (9)	0.0336 (8)	0.0416 (8)	0.0046 (7)	0.0087 (6)	0.0029 (6)
O2	0.0536 (8)	0.0376 (8)	0.0576 (9)	−0.0038 (7)	−0.0134 (7)	−0.0018 (6)
C11	0.0701 (13)	0.0399 (12)	0.0516 (12)	0.0116 (11)	0.0031 (10)	−0.0035 (9)
C12	0.0666 (15)	0.0666 (17)	0.0887 (19)	0.0219 (13)	0.0124 (13)	−0.0028 (14)
C21	0.0423 (10)	0.0295 (9)	0.0386 (9)	0.0002 (8)	0.0005 (7)	0.0011 (8)
C22	0.0479 (11)	0.0445 (12)	0.0498 (12)	−0.0086 (9)	−0.0002 (9)	−0.0006 (9)
C23	0.0613 (14)	0.0576 (14)	0.0563 (13)	−0.0077 (11)	−0.0151 (11)	−0.0097 (11)
C24	0.0732 (16)	0.0599 (15)	0.0405 (11)	0.0076 (12)	−0.0076 (11)	−0.0081 (10)
C25	0.0620 (14)	0.0667 (15)	0.0442 (12)	0.0032 (11)	0.0132 (10)	0.0001 (11)
C26	0.0444 (11)	0.0458 (12)	0.0496 (12)	−0.0047 (9)	0.0028 (8)	−0.0014 (9)

### Geometric parameters (Å, º)

**Table:**

P1—O2	1.4925 (14)	C21—C22	1.385 (2)
P1—O1	1.5529 (14)	C21—C26	1.393 (3)
P1—C11	1.792 (2)	C22—C23	1.382 (3)
P1—C21	1.7950 (19)	C22—H22	0.93
O1—H1	0.87 (2)	C23—C24	1.377 (3)
C11—C12	1.517 (3)	C23—H23	0.93
C11—H11A	0.97	C24—C25	1.363 (3)
C11—H11B	0.97	C24—H24	0.93
C12—H12C	0.96	C25—C26	1.385 (3)
C12—H12A	0.96	C25—H25	0.93
C12—H12B	0.96	C26—H26	0.93
			
O2—P1—O1	115.16 (8)	C22—C21—C26	118.40 (18)
O2—P1—C11	111.05 (10)	C22—C21—P1	121.91 (14)
O1—P1—C11	103.08 (9)	C26—C21—P1	119.69 (14)
O2—P1—C21	109.17 (8)	C23—C22—C21	120.68 (19)
O1—P1—C21	107.36 (8)	C23—C22—H22	119.7
C11—P1—C21	110.87 (9)	C21—C22—H22	119.7
P1—O1—H1	113.5 (16)	C24—C23—C22	120.06 (19)
C12—C11—P1	116.28 (16)	C24—C23—H23	120.0
C12—C11—H11A	108.2	C22—C23—H23	120.0
P1—C11—H11A	108.2	C25—C24—C23	120.1 (2)
C12—C11—H11B	108.2	C25—C24—H24	119.9
P1—C11—H11B	108.2	C23—C24—H24	119.9
H11A—C11—H11B	107.4	C24—C25—C26	120.3 (2)
C11—C12—H12C	109.5	C24—C25—H25	119.8
C11—C12—H12A	109.5	C26—C25—H25	119.8
H12C—C12—H12A	109.5	C25—C26—C21	120.43 (19)
C11—C12—H12B	109.5	C25—C26—H26	119.8
H12C—C12—H12B	109.5	C21—C26—H26	119.8
H12A—C12—H12B	109.5		
			
O2—P1—C11—C12	174.11 (16)	C26—C21—C22—C23	−0.3 (3)
O1—P1—C11—C12	50.26 (18)	P1—C21—C22—C23	179.01 (16)
C21—P1—C11—C12	−64.34 (19)	C21—C22—C23—C24	0.3 (3)
O2—P1—C21—C22	−168.19 (15)	C22—C23—C24—C25	−0.1 (3)
O1—P1—C21—C22	−42.73 (17)	C23—C24—C25—C26	−0.2 (3)
C11—P1—C21—C22	69.16 (18)	C24—C25—C26—C21	0.2 (3)
O2—P1—C21—C26	11.07 (18)	C22—C21—C26—C25	0.0 (3)
O1—P1—C21—C26	136.53 (16)	P1—C21—C26—C25	−179.28 (15)
C11—P1—C21—C26	−111.58 (17)		

### Hydrogen-bond geometry (Å, º)

**Table:**

*D*—H···*A*	*D*—H	H···*A*	*D*···*A*	*D*—H···*A*
O1—H1···O2^i^	0.87 (2)	1.64 (2)	2.4931 (19)	168 (2)

Symmetry code: (i) −*x*+1/2, *y*+1/2, *z*.
